# Bone mineral density of the femoral neck in resurfacing hip arthroplasty: Precision of DXA biased by region of interest and rotation of the hip

**DOI:** 10.3109/17453674.2010.480935

**Published:** 2010-05-21

**Authors:** Jeannette Østergaard Penny, Ole Ovesen, Kim Brixen, Jens-Erik Varmarken, SØren Overgaard

**Affiliations:** ^1^Department of Orthopedic Surgery; ^2^Department of Endocrinology, Odense University Hospital, Odense; ^3^Department of Orthopedic Surgery, Naestved Hospital, Naestved; ^4^Clinical Institute, University of Southern Denmark, OdenseDenmark

## Abstract

**Background and purpose:**

Resurfacing total hip arthroplasty (RTHA) may preserve the femoral neck bone stock postoperatively. Bone mineral density (BMD) may be affected by the hip position, which might bias longitudinal studies. We investigated the dependency of BMD precision on type of ROI and hip position.

**Method:**

We DXA-scanned the femoral neck of 15 resurfacing patients twice with the hip in 3 different rotations: 15° internal, neutral, and 15° external. For each position, BMD was analyzed with 3 surface area models. One model measured BMD in the total femoral neck, the second model divided the neck in two, and the third model had 6 divisions.

**Results:**

When all hip positions were pooled, average coefficients of variation (CVs) of 3.1%, 3.6%, and 4.6% were found in the 1-, 2-, and 6-region models, respectively. The externally rotated hip position was less reproducible. When rotating in increments of 15° or 30°, the average CVs rose to 7.2%, 7.3%, and 12% in the 3 models. Rotation affected the precision most in the model that divided the neck in 6 subregions, predominantly in the lateral and distal regions. For larger-region models, some rotation could be allowed without compromising the precision.

**Interpretation:**

If hip rotation is strictly controlled, DXA can reliably provide detailed topographical information about the BMD changes around an RTHA. As rotation strongly affects the precision of the BMD measurements in small regions, we suggest that a less detailed model should be used for analysis in studies where the leg position has not been firmly controlled.

## Introduction

Aseptic loosening is the major cause of revision of total hip arthroplasty (THA), and data from the Nordic national hip registries have demonstrated higher revision rates, up to 20% after 10 years, for younger patients (Kärrholm et al. 2008, Overgaard et al. 2008). To improve longevity, the metal-on-metal resurfacing total hip arthroplasty (RTHA) is now widely used. RTHA reduces volumetric wear (Anissian et al. 1999, Fisher et al. 2006), thought to play a prominent role in osteolysis (Ingham and Fisher 2000, Hallan et al. 2006, Howie et al. 2007). In addition, the load is thought to be naturally transferred to the proximal femur, which may prevent stress shielding (Harty et al. 2005, Hayaishi et al. 2007, Little et al. 2007, Lian et al. 2008), thereby preserving bone stock postoperatively. This mechanism should also protect the femoral neck, but there have been few prospective bone mineral density (BMD) studies focusing on the femoral neck alone. There are, however, indications that bone strain in the femoral neck of a RTHA differs from normal strain near the rim of the implant (Gupta et al. 2006), and that the entire neck area can be influenced by implant position (Vail et al. 2008) and cementation (Radcliffe and Taylor 2007). Longitudinal in vivo studies on the femoral neck are needed to determine whether RTHA preserves the bone, and if not, whether change is correlated to failure. For these prospective studies, a precise method is needed.

Dual-energy X-ray absorptiometry (DXA) is used to study BMD around standard femoral stem designs using the Gruen zones, and reliability studies have shown good reproducibility (Yamaguchi et al. 2000). In RTHA, the bone of interest is the femoral neck, which allows only rather small regions of interest (ROIs) and may contribute to reduced precision when measuring BMD (Engelke et al. 1995, Gehrchen 1999). No consensus exists on which size of ROIs to use in the femoral neck. Several models have been used (Kishida et al. 2004, Murray et al. 2005, Lian et al. 2008), but only 1 publication has reported the reproducibility (Murray et al. 2005). If region size is a factor, then we must also consider the anatomy of the neck. The anteversion means that rotations of the hip will alter the neck length of the screen image and cause a change in region size that could affect the BMD results.

We evaluated the reproducibility of BMD in the femoral neck surrounding an RTHA under 2 different set-ups: (1) the effects of increasing subdivisions/numbers of ROIs in the neck area, and (2) the effects of hip rotation on the precision of BMD measurement.

## Patients and methods

### Patients

Our sample size of 16 was based on 5% type-one error, 20% type-two error, a minimal relevant difference (MEREDIF) of 0.02 g/cm^2^, and a standard deviation (SD) of 0.02 g/cm^2^. There have been no publications linking a specific BMD loss to failure. As the clinical MIREDIF is therefore unknown, we chose to dimension our study to be able to detect a difference of the same size as the SD found in rotation studies estimating reproducibility for the intact femoral neck (Goh et al. 1995, Rosenthall 2004).

15 patients with RTHA could be included in the study, leading to a power of 78%. Permission from the Regional Ethics Committee of Vejle and Funen Counties was obtained (issued November 21, 2006; ref no. VF-20060090), and an invitation to participate in the study was sent to all 68 previously operated RTHA patients resident in Funen County, Denmark. After obtaining verbal and written informed consent, 15 patients (11 male) with a self-rated well-functioning hip were included in the study. The patients had a median age of 62 (38–73) years at the time of surgery. They were operated at Odense University Hospital from October 2005 to October 2006. Median time from surgery to DXA scan was 11 (6–18) months.

### Surgical technique and implants

The posterolateral approach was used and an ASR RTHA (DePuy, Warsaw, IN) was inserted following guidelines from the manufacturer. The components were made from a high-carbon cobalt-chromium-molybdenum alloy. The cup had an outer porous-bead coating (“Porocoat”) of hydroxyapatite (low crystalline, high purity, thickness 30–50 μm), and was placed without cement (press-fit). The femoral component was fixed with SmartSet GHV bone cement (DePuy). We aimed for a cup inclination of 45° with 20° anteversion. The pin of the femoral head was intended to be parallel with the axis of the femoral neck in the axial view and parallel or in slight valgus in the AP view. Full weight bearing was allowed after surgery.

### Scanning techniques

BMD was measured using a Hologic 4500A (Waltham, MA) DXA scanner and Hologic “metal-remove” software version 8.26A/3. Scans were performed with a resolution of 0.5 line pair/mm and a speed of 2.5 mm/sec. Radiation dosage was 0.20 mGy per examination.

**Figure 1.**
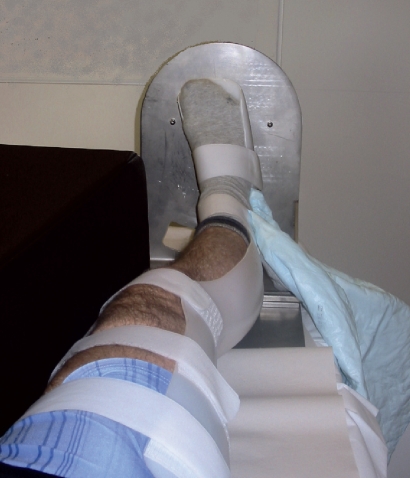
The leg strapped in the plastic shell and positioned in 15° internal rotation.

The patients were placed in supine position. The leg was strapped in a suitable-sized shell (Figure 1). The shells, custom-made from hard plastic by a prosthetic limb manufacturer (Sahva A/S, Odense, Denmark), were modeled on 4 different leg sizes (left and right leg). They went from the toes to the mid/upper thigh, with an anterior opening for entry, and were fitted with Velcro straps for circumference adjustments. The ankle and knee were reinforced for stability. The shells were designed to lock movements of the knee and foot/ankle joints, so that hip rotation could be controlled during scanning. A metal peg was mounted in the heel of the mold and fitted in an angle measurer. It could rotate the shell 45° in either direction, could be locked in any position, and was supplemented with holes and a peg for exact replication of the 15° internal, 0° neutral (toes up), and the 15° external positions. As the normal anteversion of the femoral neck ranges from 10° to 20° (Reikeras et al. 1983), we assumed that the optimal scan for a direct view of the neck would be in-between, i.e. 15° of internal rotation As this is also the standard position in most manufactured footplates, we chose to scan the femoral neck in that position. Postoperatively, however, the patients are often more comfortable with the hip in external rotation and will seek this position despite the footplate by flexing the ankle and knee. At later follow-ups, they are often able to rotate inwards. Consequently, rotation of more than 15° is likely to occur in a longitudinal study. We therefore investigated the effect of increments of rotation of 15°, from 15° of internal rotation to neutral and from neutral to 15° of external rotation, as well as the effect of a full 30° rotation from internal to external rotation.

For investigation of reproducibility, the patients were mobilized and walked around for a few minutes after the first scanning before being strapped in the shell again for the second scanning. To imitate different scanning sessions, the shell and angle measurer were detached, moved, and reattached between the 2 scans.

### Regions of interest

**Figure 2.**
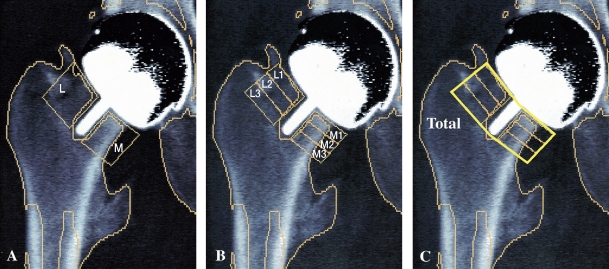
A. The 2-ROI model with a medial (M) and a lateral (L) region. The length of a region was equal to the pin length in the on-screen image. The width of the region corresponded to the distance from the pin to the rim of the femoral component. B. The 6-ROI model. The on-screen pin length was divided by 3 to create 6 subregions: 3 medial regions (M1-3) and 3 lateral regions (L1-3), again with width defined by pin and rim. C. All 6 subregions combine to make the 1-ROI (total) model.

BMD (g/cm^2^) was analyzed in 3 models: (A) in a 2-region of interest (ROI) model with a subregion medial (M) and lateral (L) to the femoral pin; (B) in a 6-ROI model as suggested by Kishida et al. ( 2004) with 3 regions medial (M1-3) and 3 regions lateral (L1-3) to the pin; and (C) in a model including the total femoral neck (total) (Figure 2). No software was available for computing the regions; a technician marked them following protocol. A new set of regions had to be marked for each position of the hip, as on-screen pin length and distance to the rim of the femoral component changed with rotation due to change in anteversion. The computer automatically summed up all the marked regions for a “total” femoral neck BMD.

### Data acquisition

For calculation of reproducibility from first to second scan, the regions marked on the first scan were copied using the “compare” mode of the computer. Copying rather than marking the model up again may give a high reproducibility in 2 repeated scans, but it reflects reality. It is common to create a permanent analyzing model at baseline. When analyzing around a THA, the software provides a Gruen zone model that is adapted to the patient’s THA. The patient-specific adaptation is saved and, via the “compare mode”, is used to analyze the following scans in a longitudinal study.

For the rotation analyses, we used the first series of scans, where we compared BMD in corresponding regions but in different rotations.

All DXA measurements were performed and analyzed according to protocol by a single trained technician who was blinded regarding the endpoint results.

### Statistics

The same bone, scanned twice a few minutes apart, should have the same BMD. If the results are close, the variation from scan to scan is little and the method is precise. As a measure of the precision of the DXA scans, standard deviations were calculated on the difference between 2 paired BMD measurements (SDdiff). To evaluate the precision between repeated measurements in the same position or different rotations, we calculated confidence intervals for the SDdiff values (Gluer et al. 1995) and compared the SDdiff values by the variance ratio tests (F-test). A 5% level of statistical significance was chosen. Thus, all statistical inference is based on the SDdiff values. To facilitate comparison with other studies, we also report coefficients of variation (CVs). The CV is a percentage-wise transformation for the precision of the BMD result. It is computed as CV = SDdiff × 100/mean BMD, and the lower the CV the more precise is the method. To give a conservative estimate of CV, the SDdiff of any rotation was compared to the higher of 2 possible SDdiff values from the repeated-measurement study.

Likewise, the rotational CVs were computed using the higher of 2 potential mean BMDs. STATA software version 9.2 (StataCorp LP, College Station, TX) was used for all analyses. A biostatistian supervised the data handling.

## Results

All BMD values (with SD), SDdiff values (with 95% CI), and CV values for the repeated measurements in different hip positions are given in Table 1.

**Table 1.** BMD, SDdiff, and CV values in 3 hip positions (repeated measurements)

	15° internal rotation			neutral (0°)			15° external rotation		
Regions	Mean BMD (SD) g/cm2	SDdiff (95% CI)	CV%	Mean BMD (SD) g/cm2	SDdiff (95% CI)	CV%	Mean BMD (SD) g/cm2	SDdiff (95% CI)	CV%
Total	0.94 (0.096)	0.028 (0.021–0.044)	3.0	0.95 (0.097)	0.024 (0.018–0.039)	2.5	0.99 (0.12)	0.037 (0.027–0.058)	3.7
*2-ROI*									
Medial	1.2 (0.10)	0.028 (0.021–0.045)	2.3	1.2 (0.12)	0.027 (0.020–0.042)	2.2	1.2 (0.13)	0.043 (0.031–0.067)	3.5
Lateral	0.73 (0.083)	0.029 (0.021–0.046)	4.0	0.75 (0.090)	0.025 (0.019–0.040)	3.3	0.80 (0.14)	0.048 (0.035–0.075) ^**a**^	6.0
*6-ROI*									
M1	1.2 (0.095)	0.056 (0.041–0.089) ^**a**^	4.6	1.2 (0.12)	0.028 (0.021–0.044)	2.3	1.2 (0.15)	0.054 (0.040–0.085) ^**a**^	4.3
M2	1.2 (0.11)	0.034 (0.025–0.053)	2.8	1.2 (0.12)	0.036 (0.026–0.056)	3.0	1.2 (0.12)	0.052 (0.038–0.081)	4.2
M3	1.2 (0.211)	0.033 (0.024–0.052)	2.9	1.2 (0.14)	0.039 (0.029–0.061)	3.2	1.3 (0.15)	0.052 (0.038–0.082)	4.1
L1	0.73 (0.094)	0.037 (0.027–0.058)	5.1	0.72 (0.086)	0.035 (0.025–0.055)	4.8	0.75 (0.097)	0.054 (0.040–0.085)	7.2
L2	0.72 (0.10)	0.044 (0.032–0.069)	6.1	0.74 (0.11)	0.039 (0.028–0.061)	5.3	0.80 (0.16)	0.052 (0.038–0.081)	6.5
L3	0.82 (0.18)	0.038 (0.028–0.060)	4.6	0.79 (0.13)	0.027 (0.020–0.043)	3.4	0.86 (0.20)	0.074 (0.054–0.12) ^**b**^	8.6

^**a**^ statistically significantly different from neutral.

^**b**^ statistically significantly different from both neutral and 15° internal rotation.

### 6-subregion (6-ROI) model

The repeated BMD measurements, with the hip held firmly in the same position, gave an average (all 3 rotations and all 6 subregions combined) SDdiff of 0.044 (0.028–0.074) g/cm^2^, corresponding to an average CV of 4.6% (2.3–8.6). The CVs in the 6-ROI subregions tended to be higher with the hip scanned at 15° of external rotation. The SDdiff values for regions M1 and L3 in the 15° external position were larger than for the neutral position (p = 0.02 and p < 0.001, respectively); the remaining regions had p-values ranging from 0.1 to 0.3. The variance was larger in the internal position than in the neutral position for the M1 region (p = 0.01) but the variation in the other 5 regions was quite similar, with p-values ranging from 0.2 to 0.8. Compared to the internal position, the variation was larger for the external position in the L3 region (p = 0.02). The remaining 5 regions generally had low p-values, but not enough to demonstrate any statistically significant difference in favor of the internal position.

### 2-region (2-ROI) model

The average SDdiff of the 2 region model was 0.033 (0.025–0.048) g/cm^2^, corresponding to a CV of 3.6% (2.2–6.0). Again, when the hip was scanned at 15° of external rotation, the lateral region had higher variation than the neutral position (p = 0.02), and was bordering on being statistically significant for the 15° internal position (p = 0.07). The medial region was unaffected by foot position.

### 1-region model (total)

When the femoral neck was analyzed as one region (total), the average SDdiff value was 0.030 (0.024–0.037) g/cm^2^, corresponding to a CV of 3.1% (2.5–3.7), and the position of the leg did not affect the variation significantly.

We observed that when we increased the number of regions in the analysis model, we detected larger variability/declining precision, but this observation was not statistically significant.

### Effects of hip rotation

Rotating the hip in increments of 15° or 30° adversely affected the variation reflected by an increasing SDdiff (Table 2), and thereby increased the CV compared to the repeated measurements.

**Table 2.** SDdiff and CV values in 3 rotational increments

	15° internal rotation to 0°		0° to 15° external rotation		15° internal to 15° external rotation	
Regions	SDdiff (95% CI) g/cm2	CV%	SDdiff (95% CI) g/cm2	CV%	SDdiff (95% CI) g/cm2	CV%
Total	0.033 (0.024–0.052)	3.5	0.054 (0.040– 0.086)	5.5	0.071 (0.052–0.112) ^**a**^	7.2
*2-ROI*						
M	0.040 (0.029–0.062)	3.3	0.024 (0.017–0.037) ^**a**^	1.9	0.055 (0.040–0.087)	4.5
L	0.053 (0.039–0.084) ^**a**^	7.1	0.088 (0.065–0.14) ^**a**^	11	0.131 (0.096–0.207) ^**a**^	16
*6-ROI*						
M1	0.070 (0.051–0.11)	5.7	0.051 (0.037–0.081)	4.2	0.095 (0.070–0.15) ^**a**^	7.8
M2	0.043 (0.031–0.068)	3.5	0.034 (0.025–0.053)	2.7	0.060 (0.044–0.094)	4.9
M3	0.17 (0.12–0.26) ^**a**^	14	0.051 (0.037–0.080)	4.0	0.16 (0.12–0.25) ^**a**^	12
L1	0.057 (0.04–0.089)	7.8	0.084 (0.062–0.13)	11	0.091 (0.067–0.14) ^**a**^	12
L2	0.059 (0.043–0.093)	8.0	0.12 (0.085–0.18) ^**a**^	15	0.16 (0.12–0.25) ^**a**^	20
L3	0.23 (0.17–0.36) ^**a**^	28	0.14 (0.10–0.21) ^**a**^	16	0.31 (0.23–0.49) ^**a**^	36

^**a**^ statistically significantly different from largest of two SDdiff values of the repeated mesurements.

### 6-region (6-ROI) model

In the 6-ROI model, the average SDdiff (based on all 3 rotation arches) more than doubled to 0.11 (0.034–0.31) g/cm^2^, corresponding to an average CV of 12% (2.7–36). The BMD measurements changed mainly in the distal and lateral parts of the neck, where the variation, even for smalll rotations, was statistically significantly larger compared to repeated measurements in the same position.

### 2-region (2-ROI) model

During rotation in the 2-ROI model, the average SDdiff (all 3 rotation arches) rose to 0.065 (0.024–0.13) g/cm^2^, corresponding to a CV of 7.4% (1.9–16), but only the lateral region was adversely affected to a statistically significant extent.

### 1-region model (total)

Compared to repeated measurements, the total femoral neck was significantly affected over a 30° rotation arch with SDdiff increasing to 0.071 g/cm2, corresponding to a CV of 7.2% (p = 0.01), but the increase in CV from 15° of internal rotation to neutral or from neutral to 15° of external rotation did not reach statistical significance.

Again, we observed that when we increased the number of regions in the analysis model we observed greater variability and declining precision.

## Discussion

We found that all models had low CVs when the hip was scanned in the same position. The 15° externally rotated position tended to be less reproducible than the others. One explanation could be that the area was slightly smaller (as seen from the higher BMD) in external rotation, leading to some difficulty in placing the ROIs.

As expected (Gehrchen 1999), we observed declining reproducibility with regions of smaller size. However, despite the fact that we subdivided the femoral neck into 6 small regions, a mean CV of 4.6% meant that DXA could detect a mean BMD change of 9% with 95% confidence in this population.

Because the CV is only slightly greater than CVs obtained from the Gruen zones around a standard THA (Cohen and Rushton 1995, Kroger et al. 1996, Sabo et al. 1998, Yamaguchi et al. 2000) and substantially better than in plain radiographs (Engh et al. 2000), it seems reasonable to use DXA in longitudinal evaluations of bone changes around an RTHA.

In contrast to the Gruen zones (Kroger et al. 1996, Mortimer et al. 1996), the femoral neck regions were highly sensitive to change in position. Rotation of the leg in increments of 15º and 30º increased the variability in all models, and had dramatic effects on the distal part of the 6-ROI model, where the CV was increased to unacceptable levels of up to 36%. Not all subregions showed statistically significant effects. It could be that there were none, but it could also be that our study was under-dimensioned, as we had a power of only 78% and those regions had change of variation below the set MIREDIF of 0.02 g/cm^2^ that our study was dimensioned to detect. That the BMD of the distal part of the femoral neck was affected the most can be explained by the dependence of precision on variation in area (Engelke et al. 1995). Over a rotation arch, this part of the bone—being furthest away from the center of rotation—would experience the largest change of area.

Rotation also affects the intact femoral neck (Wilson et al. 1991, Goh et al. 1995, Rosenthall 2004) but contrary to our findings around a RTHA, the effect is less important with CVs below 3% for the rotations found in clinical settings. As the intact-neck studies have also used a model that includes the outer margins of the neck and where area depends on the rotation, the only explanation for the exaggerated response in our models must be an added variability from the metal or removal of the metal on the scan.

Our study might have been strengthened further if we had also measured the precision achievable with the standard footrests. A precision study by Murray et al. (2005) found an overall CV of 5% using inward-rotated standard footrests modified with an extra Velcro strap, which compares well with our rigid fixation. However, Murray’s patients are well-functioning after 2-year follow-ups, presumably with no difficulty in maintaining the inward rotation. In a longitudinal study, postoperative pain and contractures could cause a resistance to the inward rotation at baseline and at the early follow-ups.

We tried to control the rotation better than with standard footrests, but we could not validate this. However, we would not have been able to do our rotation study with the standard footrest alone. To demonstrate that the femoral neck actually moves correspondingly with the shell, we could have used CT validation but refrained from that as the radiation from repeated scans could pose a risk to the patients. To support our assumption, the shells were meticulously designed to rotate in the hip alone. We asked the patients to try to move inside the shell; none could—and finally it was obvious from the appearance of the lesser trochanter on the scans that the hip joint moved with the shell. As immobilizing the knee and ankle is found to reduce the measuring error by almost half compared to standard footrests (Goh et al. 1995), we would not expect footrests to be adequate in a scanning model sensitive to rotation, but if useable, it would certainly be more comfortable for the patients and cause less effort for the staff.

The larger-region analysis models are the most precise, but they lack detail. If we had collected longitudinal scans without rigid control of the hip, we would have analyzed them using the 2-ROI model. With CVs under 5%, the medial region is robust during rotation, and would enable us to focus on the calcar bone stock in particular. The lateral region is sensitive to rotation and cannot be trusted to provide valid BMD measurements, but the computer can automatically create a “total” from the 2 regions and give us a valid overall femoral neck BMD that can allow smaller rotations. However, if planning a longitudinal RTHA trial, we suggest that the more detailed 6-ROI model should be used. It requires rigid fixation but provides regional detail, and future studies may tell us whether a particular anatomical localization of the femoral neck is crucial to the long-term survival of the prosthesis.
